# The Role of Diabetic Choroidopathy in the Pathogenesis and Progression of Diabetic Retinopathy

**DOI:** 10.3390/ijms241210167

**Published:** 2023-06-15

**Authors:** Luca Scuderi, Serena Fragiotta, Mariachiara Di Pippo, Solmaz Abdolrahimzadeh

**Affiliations:** 1 Department of Sense Organs, Sapienza University of Rome, 00161 Rome, Italy; luca.scuderi@uniroma1.it; 2 Ophthalmology Unit, Neurosciences, Mental Health, and Sensory Organs (NESMOS) Department, Sapienza University of Rome, Via di Grottarossa 1035/1039, 00189 Rome, Italy; serena.fragiotta@uniroma1.it (S.F.); mariachiara.dipippo@uniroma1.it (M.D.P.); 3 UOC Ophthalmology, Department of Surgical Areas, S.M. Goretti Hospital, 04100 Latina, Italy; 4 St. Andrea Hospital, Via di Grottarossa 1035/1039, 00189 Rome, Italy

**Keywords:** diabetic choroidopathy, diabetes mellitus, diabetic retinopathy, choriocapillaris, choroid, optical coherence tomography

## Abstract

Diabetic choroidopathy was first described on histopathological specimens of diabetic eyes. This alteration was characterized by the accumulation of PAS-positive material within the intracapillary stroma. Inflammation and polymorphonuclear neutrophils (PMNs) activation are crucial elements in choriocapillaris impairment. The evidence of diabetic choroidopathy in vivo was confirmed with multimodal imaging, which provides key quantitative and qualitative features to characterize the choroidal involvement. The choroid can be virtually affected in each vascular layer, from Haller’s layer to the choriocapillaris. However, the damage on the outer retina and photoreceptor cells is essentially driven by a choriocapillaris deficiency, which can be assessed through optical coherence tomography angiography (OCTA). The identification of characteristic features of diabetic choroidopathy can be significant for understanding the potential pathogenic and prognostic implications in diabetic retinopathy.

## 1. Introduction

Diabetic microangiopathy is one of the main responsible factors of multiorgan complications in diabetes, including nephropathy, diabetic retinopathy (DR), cardiovascular disease, and neuropathy [1,2]. The retinal vascular system, neurons, and glia constitute the neurovascular unit (NVU), where these structures present a close interdependency that promotes autoregulation, maintains the blood–retinal barrier (BRB), and provides structural support [3,4,5]. Diabetes affects the NVU, leading to retinal dysfunction and microvascular damage [6,7]. An early loss of neurovascular coupling, neurodegeneration, glial alteration, and neuroinflammation can occur even before the microvascular alterations become appreciable [3]. Although the retinal microvasculopathy in DR is preponderant and has captured most of the attention in clinical studies, the choroidal vascular layer changes are not fully elucidated [8]. The choroidal vascular bed nourishes the outer retinal layers; the foveola, in particular, relies solely on the choroid [8,9]. The retinal pigment epithelium (RPE) and the choroid appear to be affected in diabetes mellitus. The involvement of the RPE seems to be related to the breakdown of the BRB and the activation of an inflammatory response. The changes in the outer retina appear to be the result of a pathological involvement of the choriocapillaris (CC) known as diabetic choroidopathy [10].

The first evidence of choroidal involvement in DR came from histopathology, where diabetic eyes demonstrated increased arteriosclerosis and periodic-acid Schiff (PAS) positive material within the arterial and capillary walls [11]. However, the term “diabetic choroidopathy” was introduced in 1985 by Hidayat and Fine [12], who described the histopathological findings of seven eyes enucleated for late complications of diabetes. The choroidal impairment can lead to photoreceptor dysfunction and death, and CC damage can reduce the clearance of waste products from RPE cells, leading to an accumulation at the level of Bruch’s membrane (*BrM*) [13]. The systemic inflammation seems to be responsible for the choroidal damage. This is a direct result of the increased number of inflammatory cells and pro-inflammatory adhesion molecules, particularly P-selectin, which initiate the rolling on the endothelial cell surface [14].

The presence of diabetic choroidopathy was further reinforced by modern multimodal imaging techniques that demonstrated a choroidal involvement [14,15]. Particularly, optical coherence tomography angiography (OCTA) allowed qualitative and quantitative in vivo assessment of the different retinal vascular layers and the CC, through recognition of very early vascular alterations before any biomicroscopic signs [16]. Recently, CC flow deficits were found to be strongly associated with a 30 Hz flicker electroretinogram (ERG), demonstrating a direct relationship between microvascular impairment of the CC and visual function from early stages of DR on [17]. Despite this, a definitive and univocal role of diabetic choroidopathy in the pathogenesis and prognosis of DR is lacking in the literature.

The present narrative review proposes to provide an updated overview of diabetic choroidopathy, analyzing histopathological evidence, molecular alterations, and clinical correlates using multimodal imaging that define this entity in DR.

## 2. Structural Organization and Molecular Characteristics of the Choroid

### 2.1. Anatomical and Functional Organization of the Choroid

The choroidal vascular layer is supplied by the posterior ciliary arteries (PCAs), branches of the ophthalmic artery [18]. PCAs divide into branches that enter the sclera, lateral, medial, or less frequently, superior to the optic nerve. During their intraorbital course, PCAs divide into branches known as long and short ciliary arteries that do not directly arise from the ophthalmic artery and thus need to be differentiated from PCAs [19]. Long posterior ciliary arteries (medial and lateral) run radially in the horizontal meridian to reach the iris. Short posterior ciliary arteries (around 6 to 12) and the perpendicular terminal arterioles supply the innermost portion of the choroid, the CC [20,21]. More in detail, the choroid presents different vascular regions of different calibers, which include, from the innermost to the outermost layers, CC, Sattler’s, and Haller’s layer [22]. The CC is constituted of a capillary bed of fenestrated endothelial cells underneath *BrM*; the fenestration is mostly on the retinal side to allow the transit of nutrients to the RPE and the photoreceptors, but also the removal of waste products through the systemic circulation [23]. Sattler’s vascular layer is constituted of medium to small arteries, arterioles feeding the capillary layer, and veins, while Haller’s vascular layer contains large-diameter vessels of arteries and veins [22,24].

The lateral and medial PCAs supply the corresponding choroidal regions, with the medial PCA supplying the nasal choroid up to the fovea, often including the optic nerve, and the lateral PCA supplying the remaining choroidal area [18]. Each short PCA supplies a choroidal sector from the posterior pole to the equator [19], while long PCAs extend radially in the horizontal meridian, supplying small peripheral choroidal sectors posterior to the equator on the nasal and temporal sides [19]. Given its segmental nature, a compromised PCA flow can affect half of the choroidal circulation [19].

The CC has a predominant segmental organization with watershed zones, which present a particular vulnerability to ischemia or anoxia [21]. In vivo angiography studies demonstrated the CC organization in functional units characterized by lobules with a terminal arteriole and venous drainage, which do not anastomose with neighboring lobules [18,21]. The CC is more compact in the posterior pole, becoming less dense towards the periphery [19]. The venous drainage of the choroid is granted by vortex veins that exit the globe [19].

Physiologically, the choroidal circulation provides 80% of the oxygen consumed by the photoreceptors, while the remaining 20% is supplied by the retinal vasculature [25,26].

### 2.2. Immunological and Molecular Composition in the Choroidal Vasculature

The choroid is a highly active immunological site with resident inflammatory cells and continuous trafficking between the systemic circulation and the choroidal stroma [27,28]. The immune cell trafficking is mediated by a high expression of adhesion molecules at the choroidal level, which enable endothelial anchoring and stromal invasion for immunosurveillance [28,29]. CC fenestrated endothelial cells express vascular endothelial growth factors (VEGFs) 1- and 2- receptors. The CC also constitutively expresses intracellular adhesion molecule-1 (ICAM-1) that mediates the leukocytes adhesion via CD11a/CD18 or CD11b/CD18 integrins on endothelial cells [30,31]. ICAM-1, a glycoprotein that is typically expressed on endothelial cells, mostly during an immune response, is instead expressed constitutively on endothelial cells of the healthy CC [31,32].

Human leukocyte antigen (HLA) class I proteins are primarily expressed by the endothelial cells of the CC, and only minimally by the large choroidal vessels [33,34]. Different immune cell types were recognized in the choroid. Mast cells were identified in the choroid perivascularly along blood vessels of medium (Sattler’s layer) and large caliber (Haller’s layer), but preferentially distributed on the vascular walls of Sattler’s layer [35,36]. Degranulation of choroidal mast cells can be responsible for remarkable changes in the posterior segment, including choroidal thickening, choroid vessel dilation, and outer retinal barrier breakdown [37]. Resident choroidal macrophages (CD68+) were also identified in normal eyes, but without the expression of inducible nitric oxide synthase (iNOS) in *BrM* and the choroid. The expression of iNOS in activated macrophages during the inflammatory response exhibited cytotoxic and pro-angiogenic activity [38].

The enzyme carbonic anhydrase IV (CA4) was shown to be abundant in the endothelial cells of the CC, but not in the larger choroidal vessels of Sattler’s and Haller’s layer, making this one of the most specific markers of the CC [29,39,40]. Under physiologic condtions, CA4 is involved in the tranport of carbon dioxide (CO_2_) and ions contributing to the maintainance of the acid–base balance with the conversion of water and bicarbonate [39].

## 3. Pathogenetic Aspects of Choroidal Microvasculopathy in Diabetic Retina

### 3.1. Histopathological Changes

The CC represents the innermost vascular layer of the choroid, constituted of a capillary bed beneath *BrM* [9,22]. This vascular layer presents wide lumens and fenestrated endothelium surrounded by a thin basement membrane, which becomes focally or diffusely thickened in diabetic eyes. The basement membrane thickening mainly affects the scleral side of the CC close to pericytes [12]. The prominent PAS-positive basement membrane material tends to narrow and obliterate the capillary lumens throughout the choroid, with or without fibrosis. In this context, hypertrophy and hyperplasia of the endothelial cells are rare findings [12,13]. Eosinophilic PAS-positive nodules are also evident at the posterior pole and juxtapapillary region, originating from an excessive accumulation of abnormal basement membrane (BM) [12].

Two main patterns of CC degeneration are recognized using alkaline phosphatase (APase) enzyme histochemical activity. A diffuse pattern is characterized by the involvement of capillary segments without defined areas of absolute CC loss and a complete loss with a defined border of CC atrophy [13,41,42].

Capillary dropout is evident at any stage of DR, even in eyes without retinopathy or mild signs, with a topographical predilection from the temporal equator to the periphery. Capillaries with a beaded morphology and tortuosity are common features in diabetic choroidopathy [41,43]. Combining their findings with the existing literature, Cao et al. hypothesized that diabetic choroidopathy could be defined as PAS-positive material accumulation within the intracapillary stroma, often accompanied by wart-like extrusions on the vascular lumen [13].

### 3.2. The Role of Inflammation in Diabetic Retinopathy

Diabetes mellitus exhibited an inflammatory basis with increased tumor necrosis factor α (TNF-α), a pleiotropic cytokine playing a critical role in the inflammatory process and disease progression [44]. The thickening of capillary BM has been considered one of the most important events driving the microvascular damage in DR [45]. The deposition of extracellular matrix (ECM) constituents, including collagen IV, fibronectin, and laminin, contributes to the development of a thicker BM [45,46]. An altered ECM remains an early and crucial event in DR, leading to fibrosis and increased vascular stiffness and permeability [47]. The alterations at the ECM level have demonstrated to influence the leukocyte adhesion leading to leukostasis [48,49]. Inflammatory cytokines proven to induce significant changes in the expression of ECM constituents included TNF-α and IL1-β. TNF-α significantly modified the expression of collagen IV, fibronectin, agrin, and perlecan, while IL1-β influenced the expression of collagen IV and agrin [50].

Several inflammatory cytokines have been implicated in DR, including interleukins 1 (IL-1), IL-6, IL-8, TNF-α, and monocyte chemotactic protein-1 (MCP-1) [51,52]. The activation of microglia under the condition of hyperglycemia produced an increased expression of nuclear factor kappa-light-chain-enhancer of activated B cells (NF-κB), leading to increased oxidative stress and pro-inflammatory cytokines, such as IL-1b, VEGF and TNF-α, chemokines, and adhesion molecules, including E-selectin and ICAM-1 [51]. The inflammatory response with cytokines activation remains the main one responsible for the microvascular damage and cellular apoptosis in DR [53].

### 3.3. Molecular Mechanism Underlying the Choriocapillaris Loss

Inflammation appears to be a crucial element in the etiology of CC loss in diabetes. Among the activated leukocytes, polymorphonuclear neutrophils (PMNs) appear to be the leading players in diabetic choroidopathy pathogenesis. These inflammatory cells are increased in number and co-localized with areas of choroidal capillary loss, suggesting a role in the vaso-occlusive events and endothelial damage in the diabetic choroid [14,54]. Hyperglycemia seems to drive the PMN stimulation, which is in an activated state, leading to increased production and release of oxidizing agents and enzymatic exocytosis, contributing to the microvascular changes [55]. Under inflammatory stimuli, PMNs roll along the endothelium before adhering firmly to it. Activated PMNs express CD11/CD18, responsible for adhesion via binding ICAM-1 [14,56]. The interaction between PMN and endothelium is likely mediated by adhesion molecules, such as P-selectin and ICAM-1 [31,57]. At the choroidal level, ICAM-1 is expressed almost constitutively in the CC, while P-selectin is represented in postcapillary venules. The expression of these adhesion molecules is low in the retinal vessels, reflecting the difference between these two vasculatures [31,32].

The production of ICAM-1 in diabetes mellitus is also increased by nonperfusion, ischemia, and elevated TNF-α levels. The high expression of ICAM-1 on endothelial cells and elevated neutrophils represent initiating events for capillary obstruction and nonperfusion, leading to disease progression [31].

A possible explanation for the role of PMNs in producing vaso-occlusive phenomena in the diabetic choroid included the formation of queues of PMNs that could initially reduce the flow until stopping it. The accumulation of PMNs is probably due to a combination of increased leukocyte adhesion molecules and delayed blood flow [54]. The blockage of ICAM-1 via neutralizing antibodies prevents leukostasis and vascular leakage [58]. Likewise, the blockage of CD-18 impedes PMN adhesion and leukostasis [59].

Another pathogenic mechanism for diabetic choroidopathy included choroidal flow regulation. Nitric oxide (NO) is a signaling molecule produced by the vascular endothelium and involved in several physiological functions. NO synthases (NOSs) are a family of enzymes directly responsible for NO production, which include three main isoforms: neuronal NOS (nNOS), inducible NOS (iNOS), and endothelial NOS (eNOS) [60]. The nNOS and eNOS isoforms are directly involved in choroidal blood flow regulation [61]. Choroidal vasodilatation is mediated by the parasympathic system through vasoactive intestinal polypeptide, NO derived by nNOS and acetylcholine [29]. Immunoreactivity demonstrated a preferential localization for eNOS in the endothelial and smooth muscle cells of retinal arteries and capillaries, while nNOS was mainly in a subpopulation of amacrine cells. At the choroidal level, eNOS was prominently localized in the CC and less in the large choroidal vessels and stroma, and nNOS in the RPE nuclei, while iNOS was localized in the choroidal blood vessels and stroma [62]. The biological activities of NO and NOS at the choroidal level include the regulation of vascular tone, inducing vasodilatation and proangiogenic activity [62,63]. A possible role of nNOS in diabetic choroidopathy was speculated about after evidence of reduced choroidal nNOS expression 6 weeks after the development of diabetes. Choroidal nNOS is mainly expressed in the parasympathetic perivascular nerve fibers surrounding the choroidal arteries and veins, suggesting that diabetic choroidal microangiopathy can result from this imbalance [64].

## 4. Clinical and Prognostic Significance of Diabetic Choroidopathy

### 4.1. Clinical Correlates of Diabetic Choroidopathy

The abnormalities linked to diabetic choroidopathy on indocyanine green angiography (ICG) include hypofluorescent spots and small and large hyperfluorescent spots. The hypofluorescence results from dye-filling delay or CC defects associated with impaired choroidal circulation. At the same time, hyperfluorescent areas were associated with glycosylated hemoglobin values and poor glycemic control. This finding was interpreted as the clinical correlate of CC nodules, choroidal neovascularization, or intrachoroidal microvascular abnormalities seen on histopathology [65]. In eyes with nonproliferative diabetic retinopathy (NPDR), an early lobular spotty hyperfluorescent and hypofluorescent appearance (“salt and pepper”) were noted. This ICG feature was interpreted as a selective filling of the CC denoting choroidal ischemia [66]. An irregular and delayed choroidal filling on ICG was found in most patients with proliferative DR and about half of the patients with mild NPDR (background retinopathy) [67]. A salt-and-pepper pattern was found to be more frequent in eyes with advanced NPDR and proliferative diabetic retinopathy (PDR) compared to early NPDR and no DR eyes [68].

Early hypofluorescent spots, late-phase choroidal non-perfusion, inverted inflow phenomena, higher subfoveal choroidal thickness, and larger choroidal areas of non-perfusion were considered by Hua et al. [69] as potential ICG markers for the clinical definition of diabetic choroidopathy. The inverted inflow phenomenon corresponded to a delayed choroidal filling time compared to the retinal vessels [68,69]. On ultra-widefield (UWF) ICG, the advanced form of diabetic choroidopathy was described as the presence of a salt-and-pepper pattern, inverted flow phenomenon, late choroidal non-perfusion, and choroidal artery tortuosity, which the authors indicated as a sign of choroidal inflammation and increased vascular resistance [68].

Several studies have investigated choroidal thickness in DR using optical coherence tomography (OCT) with conflicting results, showing both choroidal thinning and thickening [15,69,70,71,72]. This fact can be explained by a significant choroidal thickness variability at the inter- and intra-individual levels [9]. Indeed, choroidal thickness can differ according to age, gender, and axial length among individuals [73,74,75,76,77], while at the intra-individual level, choroidal thickness can vary across different topographical locations, having the thickest point at the fovea and fluctuating during the day, with a thickening in the morning hours [78,79]. Another source of variability is represented by the OCT technology used during image acquisition [80]. The high heterogeneity and variability of the results obtained with choroidal thickness impeded identifying the potential causal relationship between choroidal changes and DR [81]. Therefore, alternative methods for studying choroidal changes through OCT have been investigated. The choroidal vascularity index (CVI) represents a more reliable estimator of the choroidal variations, as it is not influenced by other confounders [82,83,84]. This index represents the proportion of luminal area, corresponding to the vascular component, over the total choroidal area [85,86]. CVI demonstrated less variability and influence by intraocular and systemic factors than choroidal thickness. The intra- and inter-rater reliability was excellent for both the total choroidal area and the luminal component [87].

CVI was reduced in DR eyes in the macular region and even in diabetic eyes without DR compared to healthy controls [70,71,88]. The first alteration seemed to be located at the level of Haller’s layer, while Sattler’s layer was involved later as the diabetic disease progressed [71]. Alterations involving Haller’s vascular layer included focal narrowing and stumps in the choroidal vessels, which were associated with DR severity [89]. Using UWF OCT, a decreased CVI was noted in eyes with PDR. Although panretinal photocoagulation produced a choroidal thinning outside the foveal region, the CVI tended to remain lower than in healthy subjects. This finding was interpreted as a decrease in choroidal vascularity in PDR eyes [90].

Choroidal vascularity density (CVD) and choroidal vascular volume (CVV) were also evaluated as alternative indexes for evaluating the choroidal deterioration in DR. In brief, CVD can be calculated by binarizing the en-face swept-source OCT to enhance the visualization of the choroidal vasculature. In contrast, CVV was calculated by multiplying the average CVD by the macular area and the maximal choroidal thickness. Both CVD and CVV were reduced in PDR eyes, and CVD was also reduced in eyes with diabetic macular edema (DME) [91].

The introduction of OCT angiography (OCTA) allowed a better understanding of the deeper retinal vascular network and the CC [16]. The microvascular changes in DR were well evident through OCTA, also involving the CC with increasing flow deficits, as demonstrated by several authors [92,93,94,95,96,97]. When approaching the evaluation of CC in diabetic patients, it is crucial to recognize the role of confounders that may affect the CC, such as aging and hypertension [98]. This is particularly true when exploring type 2 diabetes mellitus patients, who may suffer from systemic comorbidities, impeding any possible speculation on the pathogenic role of the CC in DR [99]. Further corroborating the importance of systemic comorbidities affecting the CC, Yang et al. [97] found that the CC capillary flow density was significantly influenced by coronary artery disease, atherosclerosis, the estimated glomerular filtration rate (eGFR), glycosylated hemoglobin (HbA1c), and dyslipidemia.

Possible limitations in using OCTA for detecting early changes in the choroidal vasculature include the scanning pattern covering the macular region, considering that the most common choroidal abnormalities appeared to be localized in the mid-circumference, as well as the flow velocity in the capillaries and the medium to large choroidal vessels [100].

### 4.2. Prognostic Significance of Diabetic Choroidopathy

Choroidal abnormalities seen on ICG were associated with severe DR, poor diabetic control, and treatment. In particular, treatment influencing the choroidal blood flow, as seen on ICG, included both systemic hypoglycemic medications (insulin and oral hypoglycemic agents) and ocular intervention using panretinal photocoagulation [65,69,101]. Among OCT parameters, a larger choroidal area and choroidal thickness in Haller’s layer at baseline were associated with a higher risk of DR progression over 2 years of follow-up. The predictive value of these choroidal metrics was not influenced by other factors, such as disease duration, HbA1c, body mass index, insulin use, and mean arterial blood pressure [102]. The involvement of the outer choroidal layer was also observed in diabetic patients before starting systemic hypoglycemic medication [103].

The observation that diabetic patients without DR did not exhibit relevant differences in the choroidal vascular indices suggested the possibility that diabetic choroidopathy may be a consequence of diabetic disease rather than a factor contributing to DR pathogenesis [91]. Further, CVI tended to reduce with advanced stages of DR, particularly in the central macular region [104]. However, in the early stages of DR, a decreasing choroidal blood flow was noted, indicating primary circulatory changes in the CC before the clinical manifestations of DR [105]. Lupidi et al. [106] demonstrated CC impairment in patients with low-grade DR (Early Treatment Diabetic Retinopathy Study DR severity score of 20), postulating early damage at this level, even before the retinal microvasculature. Furthermore, CC involvement showed a moderate association with disease severity, which was more substantial for the superficial and deep retinal plexuses [94]. Interestingly, the CC alterations were recognized at any DR stage, starting from focal and/or diffuse flow defects in NPDR to large areas of severe CC impairment in PDR. Less pronounced CC abnormalities were also described in diabetic patients without DR [107].

In the initial stages of the microvascular damage, a compensatory mechanism from the deep capillary plexus (DCP) with an increasing flow to compensate for the CC insufficiency was hypothesized [106]. This hypothesis found its rationale in the capacity of the DCP in supplying the inner segments of the photoreceptors by 10–15% [108]. Retinal circulation demonstrates autoregulation in response to systemic oxygen changes with compensatory vasodilatation or constriction [109]. Diabetes induces a longstanding reduction of oxygen levels, inducing compensatory vasodilation that can be sustained to increase the blood transit to the retina, preventing the disease progression [26,110,111,112]. The choroidal circulation supplies 90% of the oxygen required by photoreceptors, creating a significant oxygen tension gradient to provide the retina with the necessary oxygen [22,113,114]. The choroidal circulation lacks autoregulation due to the high blood flow and low oxygen extraction [22]. As a result, the CC being unable to compensate for the decreasing oxygen pressure through compensatory vasodilation exhibited a progressive decline in perfusion over time [26,112].

The macular ischemia at the DCP level was associated with a legacy of outer retinal changes, including outer nuclear layer (ONL) thinning, photoreceptor disruption, and thinning [115]. Likewise, the outer retinal layer thickness was significantly influenced by CVI in eyes with DR without macular edema [70]. The analysis of the CC perfusion density in NPDR eyes demonstrated a significant association with the ellipsoid zone (EZ) “normalized” reflectivity. This parameter represents a surrogate OCT marker of photoreceptor damage obtained by post-processing the EZ en-face slabs [96]. The association between CC perfusion and EZ reflectivity in NPDR suggested that the microvascular changes in the choroid can cause photoreceptor damage and visual decline from early stages of the disease [96]. More recently, the photoreceptor status was assessed using adaptive optics (AOs), which allowed an in-depth resolution of the cone mosaic. Cone metrics, including the cone density and heterogeneity packing index, tended to reduce with increasing CC deficits of perfusion in the parafovea, while the linear dispersion index reflecting the cellular distance increased [99]. These findings were described in an NPDR population, while diabetic patients without retinopathy and healthy controls did not exhibit the same associations.

A similar study found no significant alterations in the CC parafoveal microcirculation across different diabetic patient subgroups (no DR, NPDR, and PDR) and no significant association with cone metrics on AO. The authors justified the results, suggesting that the CC may be affected by several factors, ranging from DR severity to treatment, such as pan-retinal photocoagulation and anti-VEGF medication [116]. This discrepancy further reinforces the need for an appropriate patient selection when approaching the microvascular evaluation in diabetes. In this regard, the selection of a cohort of type 1 patients without systemic comorbidities, without DR or with early signs, should be predilected [99].

## 5. Conclusions

Diabetic choroidopathy was initially described on histopathological specimens. The morphological changes included BM thickening, causing a significant narrowing of the CC capillary lumens. These alterations appeared to have an inflammatory basis, with an increased expression of adhesion molecules and PMNs that co-localize with areas of CC loss. Activated PMNs express CD11/CD18, responsible for leukocyte adhesion via binding ICAM-1. The increased expression of adhesion molecules and delayed blood flow lead to leukostasis and subsequent mechanical occlusion of the capillary lumen, causing complete obliteration and vascular dropout. Another possible hypothesis underlying the pathogenesis of diabetic choroidopathy involved NO and NOS, which are involved in vasodilatation and vascular permeability. Particularly, nNOS expressed on the parasympathetic perivascular nerve fibers of the choroid was found to be reduced in an animal model of diabetes.

The histopathological findings correlate with the structural changes found in vivo owing to the advances in retinal imaging. The choroidal alterations in vivo have been described using ICGA as early hypofluorescent spots and late non-perfusion areas, a characteristic “salt and pepper” pattern, an inverted inflow phenomenon, and choroidal artery tortuosity. Most of the studies prefer the use of non-invasive techniques, such as OCT and OCTA. The evaluation of choroidal thickness has been proven to be affected by several intra- and inter-individual confounders and thus presented conflicting results in evaluating diabetic choroidopathy. Alternative indexes obtained through imaging post-processing, such as CVI, CVV, and CVD, have been considered instead. These indexes allowed the evaluation of the different choroidal layers, particularly the medium vessels in Sattler’s and the large vessels in Haller’s layer. The latter appeared to be affected first in DR and also correlated with disease severity. The use of OCTA has permitted the evaluation of the CC and also the quantification of flow deficits. At this level, the alterations of capillary flow interest the initial DR stages, but can intervene virtually at any disease stage. Despite this, the correlation between CC deficits and DR disease severity is moderate, but the link with photoreceptor damage has been demonstrated. 

Beyond the experimental and in vivo findings, the pathogenetic role of choroidal abnormalities remains to be ascertained. Most of the literature points towards a stage-sensitive decline of the choroidal microvasculature with disease severity. However, some authors endorsed a precocious CC hypoperfusion that might play a role in the early structural modifications responsible for disease progression and visual decline. Taken together, findings from the existing literature point towards an undeniable choroidal impairment in DR, depicting and delineating the qualitative and quantitative characteristic imaging features. Diabetic choroidopathy should be considered a relevant aspect of DR, especially for the early and significant involvement of photoreceptors and, not less importantly, the functional sequelae. Knowledge of these imaging features would be auspicious to create an optimal model for studying the exact pathogenic relationship and potential therapeutic implications.

## Data Availability

No new data were created.
